# MyD88 and Its Inhibitors in Cancer: Prospects and Challenges

**DOI:** 10.3390/biom14050562

**Published:** 2024-05-07

**Authors:** Jiali Song, Yuying Li, Ke Wu, Yan Hu, Luo Fang

**Affiliations:** 1 Zhejiang Cancer Hospital, Hangzhou Institute of Medicine (HIM), Chinese Academy of Sciences, Hangzhou 310022, China; songjl@zjcc.org.cn (J.S.); wuke@zjcc.org.cn (K.W.); 2 Ruian People’s Hospital, Wenzhou Medical College Affiliated Third Hospital, Wenzhou 325000, China; layn998@wmu.edu.cn

**Keywords:** MyD88, cancer, immune escape, tumor markers, inhibitors

## Abstract

The interplay between the immune system and cancer underscores the central role of immunotherapy in cancer treatment. In this context, the innate immune system plays a critical role in preventing tumor invasion. Myeloid differentiation factor 88 (MyD88) is crucial for innate immunity, and activation of MyD88 promotes the production of inflammatory cytokines and induces infiltration, polarization, and immune escape of immune cells in the tumor microenvironment. Additionally, abnormal MyD88 signaling induces tumor cell proliferation and metastasis, which are closely associated with poor prognosis. Therefore, MyD88 could serve as a novel tumor biomarker and is a promising target for cancer therapy. Current strategies targeting MyD88 including inhibition of signaling pathways and protein multimerization, have made substantial progress, especially in inflammatory diseases and chronic inflammation-induced cancers. However, the specific role of MyD88 in regulating tumor immunity and tumorigenic mechanisms remains unclear. Therefore, this review describes the involvement of MyD88 in tumor immune escape and disease therapy. In addition, classical and non-classical MyD88 inhibitors were collated to provide insights into potential cancer treatment strategies. Despite several challenges and complexities, targeting MyD88 is a promising avenue for improving cancer treatment and has the potential to revolutionize patient outcomes.

## 1. Introduction

Myeloid differentiation factor 88 (MyD88), a key intracellular signaling cytoplasmic adaptor protein located on chromosome 3, mediates a variety of toll-like receptors (TLRs), such as interleukin-1 receptor (IL-1R) [1] and interleukin-18 receptor (IL-18R) [2,3]. As an intracellular protein, it connects the TLR family to the IL-1R-associated kinase (IRAK) family through protein–protein interactions [4]. Meanwhile, the activation of IRAK leads to multiple functional outputs, including nuclear factor-κB (NF-κB), mitogen-activated protein kinase (MAPK), and protein 1 activation, all of which make MyD88 a critical node in immunomodulation-related pathways [4]. Therefore, the transduction function of this protein has attracted extensive attention.

Recently, an increasing number of studies have shown that MyD88-driven cytokines are closely associated with cancer [5]. MyD88 induces an anti-tumor immune response, thus inhibiting tumor development [6]. On the contrary, uncontrolled innate immune signals may provide a microenvironment for cancer cell proliferation and evasion during immune surveillance, leading to further tumor deterioration [7,8,9,10]. In addition, the loss of MyD88 accelerates the development of colon tumors induced by azomethane-glucan sulfate sodium [5] and stimulates the development of skin and liver cancer [11]. Several studies have shown that MyD88-mediated signal transduction plays a key role in promoting tumor development and progression [11,12]. Based on the specificity and importance of MyD88 in regulating immunity and cancer progression, related inhibitors may provide new insights into cancer treatments. To date, there are a few narrative reviews on MyD88 that touch on aspects of inflammation and immunity interplay. However, there is no comprehensive scoping review about MyD88 on the interplay between tumor microenvironment as well as immune escape. The specific behaviors of MyD88 in regulating immune response and tumorigenesis remain unclear. Therefore, a comprehensive and unbiased overview of the current literature has the potential to identify novel themes and gaps in the knowledge base that may guide subsequent research.

This article reviews the biological function of MyD88, the key drivers affecting tumors, and the progress of research on its inhibitors. In addition, our study discusses the potential advantages and prospects of MyD88-targeted drugs in cancer therapy and proposes potential strategies for drug development and disease treatment.

## 2. Biological Structure and Function of MyD88

MyD88 is a modular protein; in the absence of a signal, inactivated MyD88 is retained in the cytoplasm. Its structure includes the C-terminal toll IL-1R (TIR) domain, N-terminal death domain (DD), and intermediate domain (INT) connecting the TIR domain and DD [13]. Previous studies have reported that N-terminal DDs can form more stable homologous and heterogeneous oligomers and spirals in solution than the weakly interacting C-terminal TIR domain [14,15]. Thus, most MyD88-dependent signaling is mediated by DD-dependent IRAK recruitment. Once MyD88 is overexpressed or binds to the downstream receptor, the DD terminus activates signal transduction and recruits the kinase IRAK [16]. Notably, overexpression of the DD terminus leads to spontaneous NF-κB and c-Jun intercellular kinase activation, even in the absence of MyD88 TIR [17]. This suggests that intracellular auto aggregation is possible even if the MyD88 TIR domain is not involved in protein interactions and is located in the DD region of MyD88. However, the N-terminus is not completely useless as it mediates interferon regulatory factor 7 (IRF-7) and promotes the production of type I interferons (IFNs) independent of DD folding [4,18]. Although this intermediate domain does not appear to be directly involved in protein interactions, it is required for activation of IRAK4 [4].

## 3. MyD88 Is Associated with Immune Inflammatory Response

TLR/IL-1R is a major family of innate pattern recognition receptors that activate wound healing and exert tissue repair mechanisms [7,19]. In TLR-mediated diseases, MyD88 is a key promoter of the signaling of almost all TLRs (except TLR3 [16]). Upon stimulation, TLR/IL-1R dimerizes and recruits MyD88, causing MyD88 to form small, unstable oligomers directly or indirectly through the Mal dimer. As the receptor continues to be activated, these oligomers continue to grow. After reaching a certain threshold, the MyD88 oligomer forms a stable interaction with IRAK4 and recruits IRAK1. Subsequently, IRAK1 phosphorylation is activated, initiating the immune signaling cascade [16].

Once the MyD88 signal is activated, this leads to a series of inflammatory response syndromes [17], which are mainly manifested by organ immune damage [20], atherosclerosis [21,22], and other immune diseases [23,24]. In addition, MyD88 can induce the expression of various chemokines and cytokines, such as tumor necrosis factor α (TNF-α), interleukin-6 (IL-6), inducible nitric oxide synthase (iNOS), cyclooxygenase 2 (COX-2), type III IFNs (λ-1, -2), and interleukin-10 (IL-10) [25,26]. With further research, an increasing number of researchers have found that inflammation is an active factor in cancer and is conducive to tumor proliferation and immunosuppression [17]. Multiple studies have shown that MyD88 has tumor-promoting effects in colorectal cancer [27], primary and metastatic breast cancer [28], Waldenstrom macroglobulinemia (WM) [29], gastric cancer [25], epithelial ovarian cancer [30], mutated diffuse large B-cell lymphoma (DLBCL) [31], breast cancer [7,32], liver cancer [33], lung cancer [34], skin cancer [7], pancreatic cancer [7], and other cancers.

## 4. MyD88 Is Associated with Tumor Progression

In human cancer, the MyD88 protein serves as a link between immune signal transduction from TLR/IL-1R and the Ras carcinogenic signaling pathway [9]. The significance of this bridging protein in tumor and immune regulation underscores its substantial potential in influencing cancer formation, proliferation, metastasis, recurrence, and prognostic regulation.

### 4.1. Abnormal MyD88 Signaling Is a Carcinogen

The most typical type of cancer induced by MyD88 is DLBCL, particularly WM. The MyD88 L265P mutation is a key driver of DLBCL development. Compared with MyD88 wild-type (WT), the TIR domain, where the MyD88 L265P mutation is located, is highly activated, resulting in enhanced downstream signaling and Myddosome complex formation. Although the MyD88 L265P mutation occurs in more than 90% of WM cases, this mutation is not WM-specific and is also observed in other DLBCLs, lymphoplasmacytic lymphomas, and a few marginal area lymphomas [35]. These results suggest that MyD88 mutation is associated with the occurrence of multiple tumors.

### 4.2. MyD88 Is Associated with Tumor Cell Proliferation and Metastasis

Vinnakota et al. found that the key regulation of TLR/MyD88 on membrane type 1 matrix metalloproteinases (MT1-MMP) was the main determinant of microglia transforming into gliomas [36]. Additionally, activation of the TLR2/MyD88 signaling pathway could activate NF-κB and Wnt signaling, participate in the pathogenesis of intracranial aneurysms [37], and induce the proliferation of colorectal cancer or breast cancer cells [9]. In breast cancer, MyD88 knockdown reduces the proliferation and migration of MCF-7 cells and increases the sensitivity of MCF-7 cells to paclitaxel [38]. This further demonstrates the importance of TLR/MyD88 in regulating cancer cell proliferation and tumorigenesis. Several recent studies have demonstrated the key role of MyD88 signaling in promoting tumor metastasis and invasion. Youn et al. [39] showed that pancreatic adenocarcinoma up-regulated factor (PAUF), a tumor-promoting protein secreted by cancer cells that acts on the TLR4 receptor on the surface of immune cells, induces human pancreatic cancer cell migration via the TLR4/MyD88/NF-κB signaling pathway rather than the TLR4/interleukin-1 receptor domain-containing adapter protein signaling pathway. Additionally, several studies have shown that MyD88 is associated with clinicopathological features, such as histological subtypes of ovarian cancer [30,40], cell metastasis of breast cancer [41], and lymphatic metastasis [42]. These findings highlight the role of MyD88 in cancer cell proliferation and metastasis.

### 4.3. MyD88 Is Associated with Tumor Prognosis

Wang et al. conducted a correlation analysis of clinicopathological variables on MyD88 expression in colorectal cancer and found that high expression of MyD88 was significantly correlated with disease-free survival and overall survival (HR: 2.33; 95% CI: 1.31–4.13; *p* = 0.0038, HR: 3.03; 95% CI: 1.67–5.48; *p* = 0.0002). They believed that MyD88 is an independent predictor of poor prognosis in colon cancer [9,43]. In ovarian cancer, TLR4-MyD88 signaling appears to be more active in high-grade cancer and is associated with reduced survival, increased recurrence, malignant transformation, and metastatic potential [44]. In high-grade serous ovarian cancer (HGSOC), high expression of MyD88 is associated with advanced stage (*p* < 0.001) cancer and moderately associated with shorter overall survival (HR: 1.13; 95% CI: 1.01–1.26; *p* = 0.04). However, in low-grade serous ovarian cancer (LGSOC), high expression of MyD88 is associated with better survival (HR: 0.49; 95% CI: 0.29–0.84; *p* = 0.009) [30]. Therefore, these results indicate that the expression level of MyD88 has different significance in different cancers; however, it could be used as evidence for predicting cancer prognosis.

### 4.4. MyD88 Could Be Considered as a Novel Tumor Marker

Previous studies have reported that the MyD88 signaling pathway is associated with carcinogenic events in various tissues and with patient survival [44]. Therefore, detection of MyD88 expression may be useful for predicting the prognosis of patients with various cancers, such as lymphoma [45], ovarian cancer [30], liver cancer [46], and colorectal cancer [9]. In an analysis of risk prognostic factors for patients with breast cancer [38], researchers found that the expression level of MyD88 in tumor tissues correlated with the stage of cancer differentiation (*p* = 0.019), and the survival rate of patients with high expression of MyD88 was lower than that of patients with low expression (*p* = 0.018). Additionally, Xiang et al. [7] found that MyD88 expression was significantly increased in highly aggressive breast cancer cells, MDA-MB-231, compared with that in normal cells and less aggressive breast cancer cells. These results indicate that MyD88 expression is associated with the proliferation and invasion of breast cancer cells and could be used as a potential target molecule in the diagnosis and treatment of breast cancer. Meanwhile, in a preclinical study of colorectal cancer, researchers found that silencing MyD88 in SW480 (human colon adenocarcinoma cells) and HCT116 (human colon cancer cells) interferes with the MyD88/NF-κB/AP-1 signaling pathway and significantly inhibits cancer cell growth and invasion. These results suggest that MyD88 activation plays an important role in inducing the development of colorectal cancer and could be used as a potential novel biomarker for colorectal cancer [27,47].

Simultaneously, Guo et al. identified the core genes involved in the regulation of the glioma immune microenvironment with the help of bioinformatics technology and found that MyD88 could cause disorders in tumor-infiltrating immune cells, especially promoting the transformation of macrophages into the M2 type [48], thereby providing good prognostic value for patients with glioma.

Additionally, calcycin-binding protein and Siah-1 interaction protein (CacyBP/SIP) is a polyligand protein that is overexpressed in cancers and associated with poor prognosis [49]. Recent studies have reported that MyD88 is a novel downstream substrate of CacyBP in hepatocellular carcinoma. Inhibition of the binding of CacyBP-MyD88 could reduce fractalkine (CX3CL1) secretion and thus weaken the recruitment of tumor-associated macrophages (TAMs) to the immune microenvironment, making hepatocellular carcinoma mice sensitive to programmed death 1 (PD-1) treatment [50]. MyD88 also serves as a target of miR-155-3p. According to a recent report, MiR-155-3p is associated with clinicopathological markers, tumor subtypes, and low survival rates in various tumor tissues [7]. Therefore, MyD88 may play a critical role in cancer progression by acting as a novel tumor marker and altering the tumor immune microenvironment, signaling, and binding of substrate proteins.

## 5. MyD88-Mediated Tumorigenic Pathway

Studies have reported that 20% of cancer cases worldwide are caused by chronic inflammation. The longer the duration of inflammation, the higher the risk of cancer [51]. MyD88 is widely involved in tumor formation and is a key promoter of inflammatory development. It activates the B-cell signaling pathway of the hematopoietic chamber and tumor cells, inducing an inflammatory environment conducive to carcinogenesis [7]. In contrast, it mediates the optimal activation of the rat sarcoma virus/extracellular signal-regulated kinase (Ras/ERK) pathway, binds to ERK, and protects it from dephosphorylation [7]. Optimal activation of the Ras/ERK pathway is crucial for the expression of DNA repair enzymes such as ERCC1, which can help cancer cells to repair damaged DNA. Therefore, inhibition of MyD88 promotes the accumulation of DNA damage, which leads to cell death via the tumor suppressor protein p53 [52]. In addition, the TLR2/MyD88 signaling pathway can activate the NF-κB and Wnt signaling pathways, ultimately inducing the proliferation of colorectal and breast cancer cells [9]. Therefore, reducing the expression or activity of TLR2 and MyD88 in the intestinal epithelium may significantly reduce the tumorigenicity of ulcerative colitis and epithelial cells.

## 6. MyD88 Is Involved in Tumor Immune Escape

**Figure 1:**
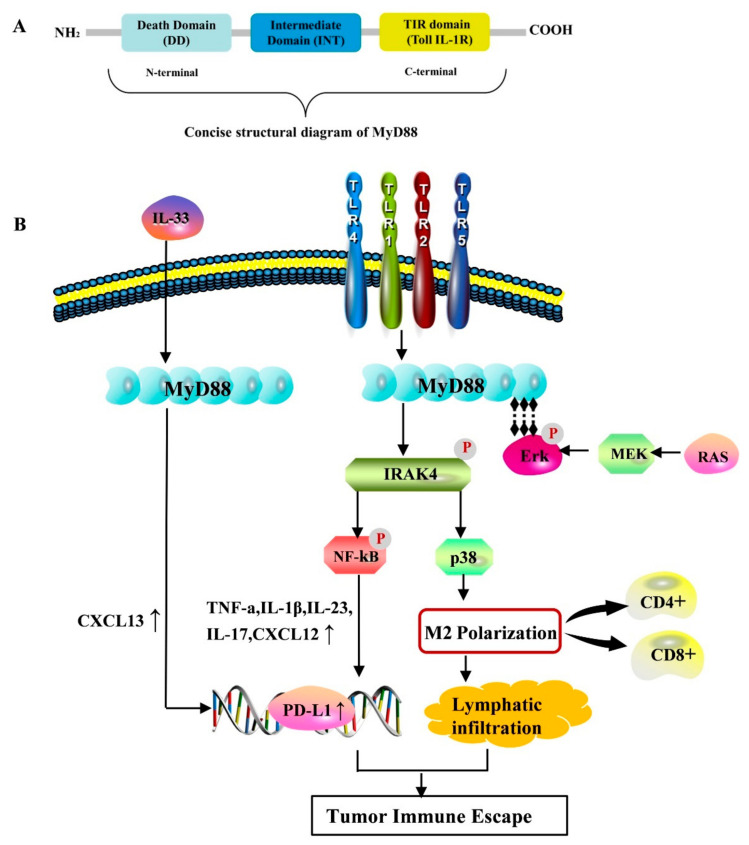
MyD88 is associated with immune escape. (**A**) Concise structural diagram of MyD88. (**B**) MyD88 is associated with immune escape. MyD88, myeloid differentiation factor 88; Erk, extracellular signal-regulated kinase; MEK, mitogen-activated extracellular signal-regulated kinase; RAS, rat sarcoma virus; IRAK4, interleukin-1 receptor-associated kinase 4; NF-κB, nuclear factor-κB; TNF-α, tumor necrosis factor α; IL-1β, interleukin-1β; IL-23, interleukin-23; IL-17, interleukin-17; PD-L1, programmed death ligand 1; CXCL12, chemokine (C-X-C motif) ligand 12; CXCL13, chemokine (C-X-C motif) ligand 13; and CD4+, cluster of differentiation 4+.

Inflammation creates conditions for tumor progression and metastasis and provides a favorable environment for tumor immune escape [7]. Increasing evidence supports the idea that immunosuppressive cells in the tumor immune microenvironment are important factors in tumor proliferation and invasion. These cells include macrophages, myeloid suppressor cells (MDSCs), dendritic cells (DCs), natural killer cells, eosinophils, and regulatory T cells [53]. Therefore, our understanding of MyD88-related tumor immune escape, promotion of M2 macrophage expression, and tumor cell DNA damage repair is slowly increasing (Figure 1).

As one of the most abundant immune cells in the TME, the transformation of M1 to M2 polarization of macrophages could affect tumor proliferation and immune escape and accelerate tumor progression, which is commonly observed in glioma [48] and pancreatic cancer [54]. Studies have also found that MyD88 is a pivotal gene in the TME and that its expression is positively correlated with M2 macrophages and negatively correlated with monocytes. Yuan et al. [55] analyzed the number of CD206+ (an M2 marker) and CD86+ (an M1 marker) macrophages in colon tissue and found that Fib-MyD88-KO mice showed an increase in the M1 phenotype and a decrease in the M2 phenotype. Meanwhile, inhibition of MyD88 can effectively slow the progression of colitis to colon cancer [55]. Additionally, studies have confirmed that MyD88 regulates M1- and M2-related genes, vascular endothelial growth factor expression, and macrophage M2 polarization through the MyD88-JAK2/TYK2-STAT3 pathway [56]. These results further demonstrate the significant role of MyD88 in promoting the M1/M2 state transformation of tumor-associated macrophages. Currently, the depletion of M2-like cells and an increase in the M1/M2 ratio are favorable strategies for anti-cancer therapy. Given the positive correlation between MyD88 and M2-macrophages, inhibition of MyD88 may be a promising anti-cancer therapeutic strategy [57].

MDSCs are precursors of DCs, macrophages, and granulocytes and are one of the most abundant immune cells in the tumor microenvironment (TME) [53]. In the MDSC-mediated tumor immune microenvironment, inhibition of MyD88 not only directly inhibits the differentiation of MDSCs but also indirectly inhibits MDSC expansion by reducing the secretion of cytokines. Additionally, a recent study found that TJM-2010-2, a novel MyD88 inhibitor, reduced the expression of iNOS, Arg-1, and the immunosuppressive molecule Indoleamine-2,3-Dioxygenase (IDO) by inhibiting MyD88, thus weakening tumor immune escape [32]. This study demonstrated that the MDSC-mediated immune escape mechanism depends on the activation of MyD88 signaling. Transgenic technology based on blocking MyD88 signaling to inhibit MDSC has successfully restricted the growth of lung and ovarian tumors in mice [53].

Additionally, programmed death ligand 1 (PD-L1) has been recognized to be deeply involved in immune escape. Studying PD-L1 helps to identify the mechanism of immunosuppression in tumor cells or antigen-presenting cells. As a critical junction molecule, MyD88 is closely associated with PD-L1 expression. It has been found that the MyD88-dependent pathway activates NF-κB conduction and induces PD-L1 transcription, assisting tumor cells to participate in immune escape. High mobility group box-1 protein (HMGB1) is a non-histone chromosomal protein involved in the regulation of tumor autophagy and apoptosis [58]. When HMGB1 is released from dying cells, the activated TLR4/MyD88 pathway enhances INF-β signal transduction [59], stimulates immune activity [60], and up-regulates the expression of PD-L1 in neighboring surviving tumor cells. In addition, the upregulation of PD-L1 expression in dendritic cells, fibroblasts, and plasma cells is mediated by TLR(1,2,4,5)/MyD88 [61], which ultimately induces lymphatic infiltration [62]. Tumor cell-released autophagosome is a type of LC3-II double-membrane extracellular vehicle (EV) that can transform macrophages into M2 macrophages. Recent studies have shown that macrophages M2 polarization relies on TLR4-MyD88-p38-STAT3 signal transduction, which effectively inhibits the proliferation of cluster of differentiation CD4+ and CD8+ T cells in vitro and promotes tumor growth in vivo, mainly through PD-L1 [63].

Additionally, the expression of PD-L1 is also regulated by related stimulators [64], such as chemokine (C-X-C motif) ligand 12, CX3CL1, interferon-gamma (IFN-γ), TNF-α, interleukin-1β, and IL-6. Notably, sustained expression of MyD88 inhibits CX3CL1 promoter histone deacetylation, thereby promoting CX3CL1 transcriptional activation and secretion, which further increases the recruitment of TAMs to the TME, inducing immune escape and anti-PD-1 tolerance. Additionally, IFN-γ [65] is a key driver of PD-L1 expression in host tumors. Some researchers have found that the activated MyD88/TRAF6/NF-κB signaling pathway not only causes upregulation of IFN-γ receptor expression, but also indirectly enhances IFN-γ-induced PD-L1 expression [66]. These studies indicate that MyD88 is critical in regulating T cell activation, macrophage polarization, and PD-L1 expression in tumor immunosuppression.

## 7. Drug Therapy Targeting MyD88

Numerous natural and synthetic products have demonstrated anti-inflammatory and anti-cancer activities that target MyD88. Based on their function, they can be divided into two modes affecting the upstream and downstream signaling of MyD88 (Table 1) and targeting MyD88 autopolymers or heteropolymers with other proteins (Table 2).

For example, Salvia miltiorrhiza mitigates cardiac dysfunction and inflammation in rats with heart failure by inhibiting the formation of the MD2/TLR4-MyD88 complex [67]. Additionally, Astragaloside IV, Xiang Lin pills, berberine, and fluoxetine can inhibit the TLR4/MyD88/NF-κB signals, thereby exerting the effects of acute myocardial infarction, colitis, and ischemia-reperfusion injury, and relieve postoperative cognitive dysfunction and neuroinflammation in elderly mice [67,68,69,70,71,72,73,74,75,76,77,78,79,80]. Polyene phosphatidylcholine, a well-known hepatoprotective drug, has also recently been found to act on the TLR2/MyD88 signaling pathway, inhibiting the binding of MyD88 and TLR, and activating the IκB kinase (IKK) complex to alleviate synovial inflammation [81]. In addition, studies have reported that pioglitazone can reduce oxidative stress through the TLR4/MyD88/NF-κB signaling pathway, thereby ameliorating the testicular toxic side effects of cisplatin treatment [75]. In addition to being anti-inflammatory, other MyD88 inhibitors have shown unexpected anti-cancer effects. For example, icariin can delay cervical cancer progression by inhibiting the TLR4/MyD88/NF-κB and Wnt/β-catenin pathways [82]. Curcumin inhibits the expression of TLR4/MyD88 and EGFR in a dose- and time-dependent manner, thereby inhibiting the invasion and migration of non-small cell lung cancer [83]. Tomisetron, a receptor antagonist primarily used for anti-emesis, has also been shown to inhibit the development of colorectal cancer by inhibiting inflammation in colitis and TLR4/MyD88 signaling [84]. A recent study reported that the combination of zoledronic acid and thymosin α1 [85] successfully treated non-immunoreactive patients with advanced or metastatic prostate cancer (PCa) by blocking MyD88/NF-κB signaling in PCa cells. This treatment activates the MyD88 signaling axis in macrophages and T cells, leading to increased infiltration of cytotoxic T cells and increased tumor inflammation. Sijunzi decoction has also been found to regulate the TLR4/MyD88/NF-κB signaling pathway and reduce the expression of PD-L1 to inhibit the growth of lung cancer [86]. MGN1703 [87] is a clinical trial drug that acts on the TLR9/MyD88 signaling axis for the treatment of metastatic colorectal cancer [88]. Good phase I/II data have been obtained for MGN1703 as a first-line maintenance drug for the treatment of HIV-1 infection after chemotherapy [89]. Additionally, researchers have found that a novel extracellular vesicle-like ginseng derived nanoparticle could induce the polarization of M1-like macrophages in mice with B16F10 melanoma through the TLR4/MyD88 signaling pathway and enhance the production of total reactive oxygen species to induce melanoma cell apoptosis [57].

**Table 1:** Inhibitors affecting MyD88 signaling (ranked according to the year the inhibitor was first reported).

Inhibitor	Structure	Machine	Effect	Year of Publication
MGN1703	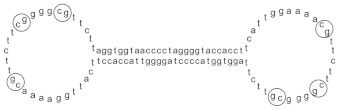	Acts on TLR9/MyD88 signaling pathway	Benefits the treatment of metastatic colorectal cancer [88], enhancing anti-viral immune response to chronic HIV-1 infection [89]	2015
Tomisetron	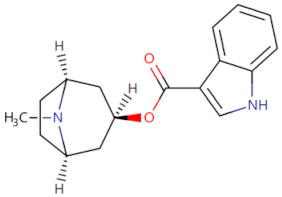	Inhibits TLR4/MyD88 signaling	Inhibits the development of colorectal cancer [84]	2016
Curcumin	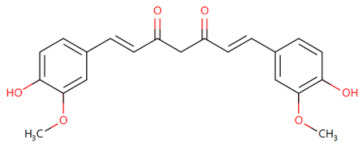	Inhibits the expression of TLR4/MyD88 and EGFR in a dose- and time-dependent manner	Inhibits the proliferation and migration of NSCLC [83]	2019
Mesalazine	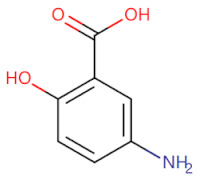	Inhibits the TLR4/MyD88-dependent pathway	Resists ulcerative colitis in mice model [76]	2019
Salvia miltiorrhiza	Compound of traditional Chinese Medicine	Inhibits MD2/TLR4-MyD88 complex formation and signaling	Reduces cardiac dysfunction and inflammatory response in heart failure rats [67]	2020
Radix gentianae	Compound of traditional Chinese Medicine	Inhibits the galectin-3/TLR4/MyD88/NF-κB inflammatory signaling pathway	Prevents acute myocardial infarction induced by isoproterenol in rats [68]	2020
Oxyberberine	Compound of traditional Chinese Medicine	Inhibits the TLR4-MyD88-NF-κB signaling pathway and the translocation of NF-κB p65 from cytoplasm to nucleus	Anti-colitis effect [90]	2020
Dexmedetomidine	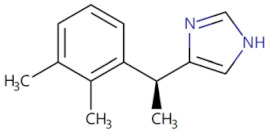	Inhibits the TLR4/MyD88/NF-κB signaling pathway	Resists Intestinal Ischemia-Reperfusion Injury [73]	2021
Rifampicin	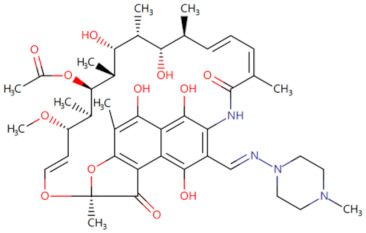	Inhibits the TLR4/MyD88/NF-κB signaling pathway	Ameliorates lipopolysaccharide-induced cognitive and motor impairments in mice [77]	2021
Astragaloside IV	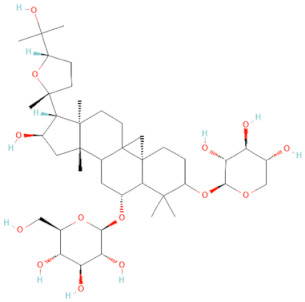	Inhibits the TLR4/MyD88/NF-κB signaling pathway	Prevents acute myocardial infarction [70]	2021
Icariin	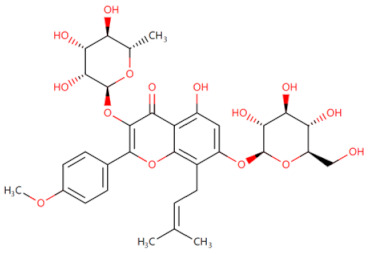	Inhibits the TLR4/MyD88/NF-κB and Wnt/β-catenin signaling pathway	Inhibits the progression of cervical cancer [82]	2021
Sevoflurane	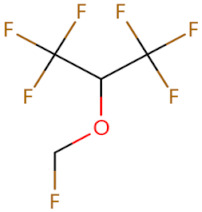	Inhibits the TLR4/MyD88/TRAF6 signaling pathway	Sevoflurane postconditioning ameliorates cerebral ischemia-reperfusion injury in rats [78]	2022
Polyene Phosphatidylcholine	—	Acts on TLR2, reduction of the activation of MyD88 IKKs complex	Inhibits the synovial inflammation induced by LPS [81]	2022
Emodin	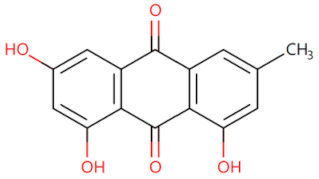	Inhibits the MyD88/PI3K/Akt/NF-κB signaling pathway	Inhibits the activation of microglia and inflammatory response [79]	2022
Biogenic AgNPs	—	Targets the TLR4/MyD88 and Nrf2/HO-1 signaling pathways	Inhibits LPS-induced neuroinflammation [71]	2023
Fluoxetine	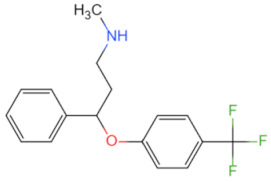	Attenuates the TLR4/MyD88/NF-κB signaling pathway activation	Alleviates postoperative cognitive dysfunction in aged mice [72]	2023
Salvianolic acid A	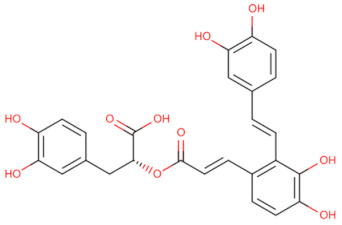	Inhibits TLR2/TLR4-mediated MyD88 activation and its downstream molecules TRAF6 and IRAK4	Attenuates cardiac inflammation and cardiac disfunction in heart failure mice [80]	2023
Atorvastatin	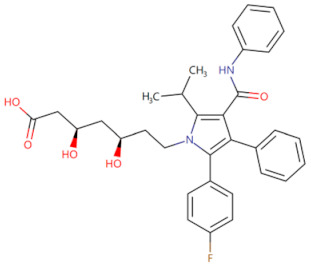	Inhibits the TLR4/MyD88/NF-κB signaling pathway	Reduces contrast media-induced proptosis of renal tubular epithelial cells [74]	2023
Pioglitazone	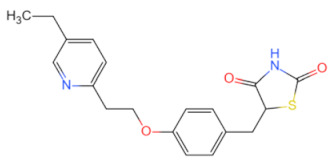	Attenuates oxidative stress and inflammation via the TLR4/MyD88/NF-κB signaling pathway	Ameliorates cisplatin-induced testicular toxicity [75]	2023
Xianglian Pill	Compound of traditional Chinese Medicine	Inhibits the TLR4/MyD88/NF-κB signaling pathway	Attenuates ulcerative colitis [69]	2023
Combined treatment with ZA and Tα1	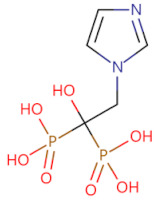 Structure of ZA	Blocks MyD88/NF-κB signaling in PCa cells and activates the MyD88/NF-κB signaling in macrophages and T cells, leading to increased cytotoxic T cell infiltration and enhanced tumor inflammation	Alleviates the non-immunoreactive patients with advanced or metastatic prostate cancer [85]	2023
Si jun zi tang	Compound of traditional Chinese Medicine	Regulates the TLR4/MyD88/NF-κB signaling pathway and reduces PD-L1 expression	Inhibits the proliferation and migration of lung cancer cells and reduces the expression of PD-L1 protein in A549 cells [86]	2024

Currently, numerous inhibitors target the signaling pathways of MyD88. However, polymerizing inhibitors that directly target MyD88 may have more precise anti-inflammatory and anti-cancer effects. Since then, a series of MyD88 targeted inhibitors have been discovered and further explored (Table 2). Currently, most reported inhibitors are small-molecule compounds that block MyD88 homopolymers or heteropolymers. Given the crucial role of the MyD88 BB loop in protein–protein interactions, the earliest developed MyD88 inhibitor was a short peptide directly derived from the BB loop (Ac-RDVLPGT-NH_2_) [91]. However, due to the inability of peptides to be orally administered in mammals and their low bioavailability, researchers developed a mimetic peptide compound AS-1, which was found to inhibit the interaction between IL-1R and MyD88 and weaken the binding activity of NF-κB [92]. Later, based on Ac-RDVLPGT-NH_2_, Dishon, S. et al. developed a novel targeted inhibitor MyD4-4 to improve experimental autoimmune encephalomyelitis in mice [93]. With the continuous development of targeted inhibitors, ST2825 has been found to be an effective inhibitor that prevents MyD88 dimerization and inhibits NFκB activation. Current research on ST2825 is not limited to the treatment of diseases related to immune inflammation (such as neuroprotection, synovitis, and arthritis in mice with traumatic brain injury), and attempts have been made to use it in the field of anti-tumor therapy, such as inhibiting the proliferation of hepatocellular carcinoma cells [94] and anti-pancreatic ductal adenocarcinoma [95]. More importantly, it can also inhibit the growth of lymphoma and leukemia cells [96], overcoming the limitations of the BTK inhibitor, ibrutinib, in the treatment of non-mutant MyD88 lymphoma and providing new treatment ideas for WT MyD88 lymphoma. Another series of small-molecule MyD88 inhibitors, TJ-M2010-X (X = 5, 2, 6), has also been reported to interfere with the homologous dimerization of MyD88. TJ-M2010-5 binds to the MyD88 TIR domain, blocks TLR/MyD88 signaling, and plays a significant role in reducing inflammatory effects and liver fibrosis [97], alleviating organ ischemia/reperfusion injury [98], preventing colitis-associated colorectal cancer [99], combating hepatocellular carcinoma [100], reducing allogeneic rejection [101], and enhancing allogeneic transplantation tolerance [102]. It can also block the TLR7/MyD88/NF-κB and TLR7/MyD88/MAPK signaling pathways and correct R848-induced lupus-like immune disease in B cells [103]. TJ-M2010-6 has been reported to prevent and treat type 1 diabetes in non-obese diabetic mice [104]. TJ-M2010-2 affects the MyD88/GSK-3β and MyD88/NF-κB signaling pathways and inhibits the proliferation, migration, and invasion of breast cancer cells [32]. Other research has led to the discovery that T6167923 [105], LM9 [106], 4210 [107], LM8 [108], M20 [109], and C17 [110] exert anti-inflammatory, anti-cancer, and anti-viral effects by inhibiting the formation of MyD88 oligomers and reducing the production of inflammatory and fibrotic factors. However, current research on MyD88-targeted inhibitors is still in the preclinical stage, and many inhibitors are used as adjunctive therapy for diseases; therefore, we need to further evaluate the challenges of these inhibitors in disease treatment.

**Table 2:** Inhibitors targeting MyD88 polymorphism (ranked according to the year the inhibitor was first reported).

Inhibitor	Structure	Machine	Effect	Year of Publication
linear RDVLPGT	—	Inhibits MyD88 dimerization	Inhibits the NF-κB signal pathway [91]	2008
AS-1	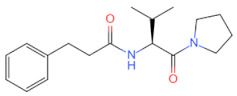	Inhibited the interaction between IL-1R and MyD88 and weakened the binding activity of NF-κB	Protects the myocardium from ischaemia/reperfusion injury [92]	2009
4210	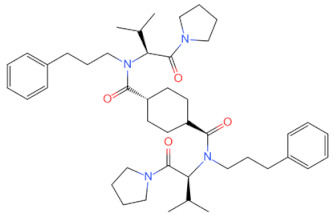	Inhibits MyD88 dimerization	Inhibits the pro-inflammatory immune signaling induced by bacterial toxins [107]	2015
		Inhibits the interaction between MyD88 and IRF3/IRF7 and upregulated IFN-α	Anti-viral effect [111]	2020
T6167923	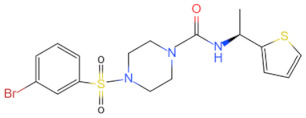	Disrupts the formation of MyD88 homodimer	Protects mice from toxic shock induced by SEB [105]	2015
		Inhibits MyD88 expression	Down-regulates the expression of Col I, Col III, and α-SMA [112]	2023
TJ-M2010-5	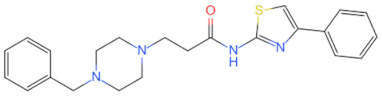	Inhibits MyD88 homologous dimerization	Prevents colorectal cancer related to colitis [99], increases the tolerance of allogeneic transplantation in mice [102], prevents DSS-induced acute liver injury in mice [113] and reduces transplant rejection in mice [101]	2015–2019
		Inhibits the MyD88 signaling pathway	Anti-hepatocellular carcinoma [100]	2019
		Inhibits the TLR7/MyD88/NF-κB and TLR7/MyD88/MAPK signaling pathways	Relieves B cell lupus-like immune disease [103]	2020
		Inhibits MyD88 homologous dimerization	Reverses myocardial ischemia and reperfusion injury [114]	2020
		Inhibits the MyD88/NF-κB and Erk pathways Inhibits myeloid cell infiltration and microglia activation	Relieves acute cerebral ischemia-reperfusion injury [115]	2022
		Inhibits MyD88 homologous dimerization	Alleviates myocardial ischemia/reperfusion injury during heart transplantation [98]	2022
		Blocks the activation of MyD88/NF-κB	Alleviates liver fibrosis [97]	2022
		Overcomes the inhibitory function of myeloid suppressor cells	Prevents colorectal cancer development associated with colitis [53]	2022
		Inhibits cell proptosis	Prevents liver ischemia-reperfusion injury [116]	2023
		Blocks MyD88 signal transduction	Prevents the development of CAC as well as downregulating GPNMB mRNA [117]	2023
ST2825	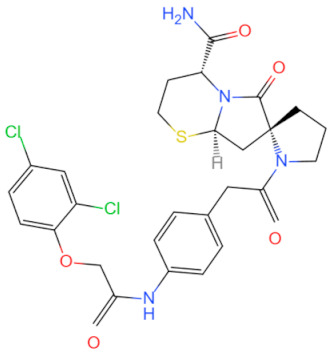	Prevents MyD88 from dimerizing	Provides neuroprotection after experimental traumatic brain injury in mice [118], inhibits HCC cell proliferation [94] and relieves lymphoma and leukemia [96]	2016, 2017
		Inhibits the NF-κB signaling pathway Inhibits the production of IL-10 and IFN-β	Relieves B-cell neoplasms driven by activated MyD88 signaling [119]	2019
		Prevents MyD88 from dimerizing	Prevents synovitis and joint degeneration [120]	2021
		Inhibits NF-κB activation and the ROS/NLRP3 signaling pathway	Attenuates LPS-stimulated neuroinflammation [121]	2022
		Prevents MyD88 from dimerizing	Inhibits pancreatic ductal adenocarcinoma [95] and alleviates synovial lesions [122]	2023
TJ-M2010-6	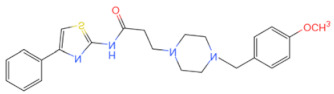	Inhibits MyD88 homologous dimerization	Prevents and cures type 1 diabetes in NOD mice [104]	2016
TJ-M2010-2	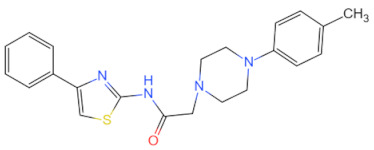	Inhibits MyD88 homologous dimerization	Offsetting renal ischemia-reperfusion-induced renal injury [123] and alleviates renal interstitial fibrosis [124]	2016, 2018
		Regulates the MyD88/GSK-3β and MyD88/NF-κB signaling pathways	Inhibits the proliferation, migration, and invasion in breast cancer cells [32]	2020
Cyclic c (MyD 4-4)	—	Prevents MyD88 from dimerizing	Alleviates immune encephalomyelitis in mice [93]	2018
LM9	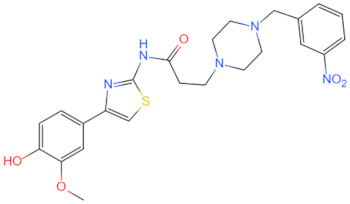	Inhibits the MyD88 and inflammatory pathways in macrophages	Prevents atherosclerosis [125]	2019
		Inhibits the formation of TLR4/MyD88 complexes	Reduces inflammatory response and cardiac fibrosis [106]	2020
LM8	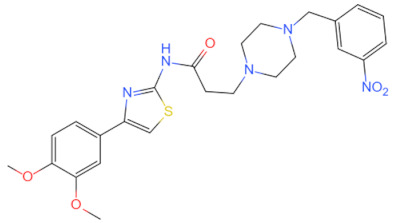	Inhibits the MyD88 Erk/NF-κB-dependent inflammatory pathway	Relieves heart damage [108]	2020
		Inhibits TLR4-MyD88 interaction and NF-κB activation	Protects the kidney from inflammatory damage of diabetes [126], relieves heart inflammation in diabetes mice [127] and relieves hypertensive nephropathy-induced by angiotensin II [128]	2021, 2022
M20	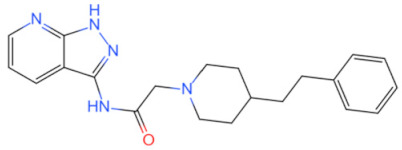	Prevents MyD88 from dimerizing	Reduces sepsis-mediated acute lung injury [109]	2021
Low temperature oxygenation perfusion combined with TJ-M2010-5	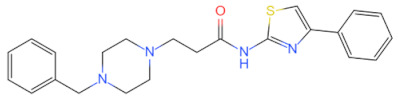	Inhibits the TLR/MyD88 signaling pathway	Relieves liver ischemia-reperfusion injury [129]	2022
C17	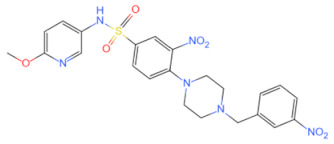	Inhibits the interaction between TLR4-MyD88 and NF-κB signaling pathway	Alleviates acute lung injury [110]	2023

**Figure 2:**
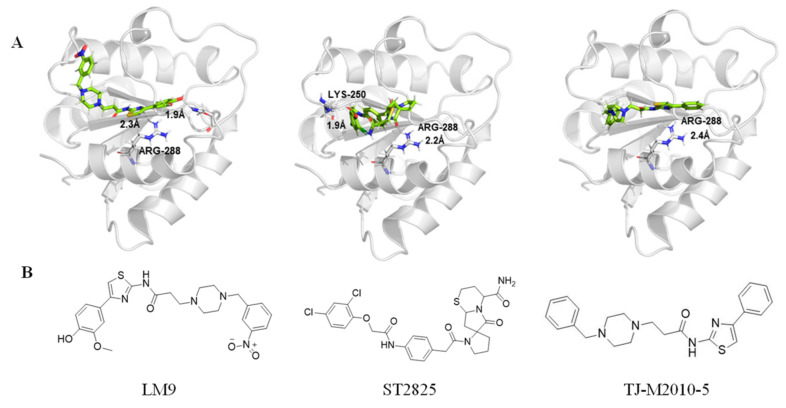
The binding site of MyD88 with inhibitors. (**A**) Binding mode of inhibitors with MyD88 TIR domain (PDB:7L6W). (**B**) Typical inhibitors of MyD88 TIR domain. MyD88, myeloid differentiation factor 88; TIR, toll interleukin-1 receptor.

Currently, the co-crystallization structure of MyD88 and its inhibitors have not been reported; therefore, it remains a mystery as to which binding site of this protein the inhibitor interacts with. Moreover, the allosteric process of Myddosome complex during signal transmission has not been detailed. We used artificial intelligence-based docking for docking analysis [130] of MyD88 with three widely reported inhibitors and found that the binding site was consistent with that reported by Clabbers et al. [131]. Residue ARG288 formed a hydrogen bond with the inhibitor, revealing a possible binding site between the protein and the inhibitor to a certain extent (Figure 2). Hence, future research could explore additional binding modes based on this site and develop more potent anti-tumor targeted inhibitors.

## 8. Discussion

As a critical cytoplasmic signaling protein, MyD88 is involved in mediating innate immunity and regulating the TME. Activated TLR/MyD88 signals can induce the secretion of immune factors and enhance the antigen-presenting ability of DCs, thereby promoting the transformation of inflammatory cancers. Through the TLRs/MyD88 signaling pathway, MyD88 directly or indirectly affects the secretion of a variety of downstream immune factors [25,26]. This induces changes in the type, quantity, and function of local immune cells in tumor tissues, leading to the deterioration of the inflammatory microenvironment and remodeling of the TME, ultimately causing tumor immune tolerance and immune escape. Additionally, based on optimal activation of Ras/ERK, MyD88 promotes DNA damage repair and self-protection in cancer cells. Considering the critical driving force of MyD88 in cancer, targeting MyD88 may be a powerful strategy in the field of anti-tumor therapy.

In this review, we gathered critical evidence of MyD88 in regulating tumors and immune microenvironment, summarize recent research progress on MyD88 inhibitors, and reveal that residue ARG288 is highly likely to be an anti-tumor binding site between Myd88 protein and inhibitors. This provides a clear and feasible approach for targeted treatment of tumors with MyD88 in the future. Meanwhile, the critical evidence concerning the regulation of MyD88 in the tumor immune microenvironment has revealed that MyD88 not only regulates inflammatory signaling pathways, but also facilitates tumor immune evasion, macrophage polarization, and stimulates tumor cell proliferation and metastasis.

Recently, with the development of structural biology and biochemical research, targeting MyD88 has become a viable therapeutic option. The reported inhibitors are mainly based on the inhibition of MyD88 homologous oligomers or heteropolymers with other proteins. In addition, some inhibitors reduce MyD88 levels by silencing genes, thus blocking MyD88 signaling. Currently, inhibitors that affect MyD88 signaling and disrupt Myddosome assembly are effective and feasible in different disease models. However, there are also some urgent issues that need to be explored, such as the small binding effect of existing inhibitors on target proteins, mainly due to the relatively flat surface of the MyD88 protein and shallow inhibitor binding pockets. In theory, the application of inhibitors with smaller chemical structures may be able to prolong the relative action time and enhance the effect of action.

In this review, there are still some limitations in the research. We summarized the progress of targeted multimerized-inhibitors in recent years, but due to the bias of literature retrieval, we did not exhaustively list all the progress on inhibitors of the MyD88 signaling pathway. At the same time, more in-depth discussions on high-expression cancer have not yet been conducted, such as ovarian cancer, which will be further explored in in subsequent studies.

In conclusion, the development of more effective inhibitors is worth considering. Although the regulation of MyD88 contributes to controlling the development of related diseases, very few inhibitors have been successfully implemented in the clinical setting. Some inhibitors have low bioavailability, short half-lives, and poor pharmacokinetic performance, which has led to many MyD88 inhibitor studies remaining in the preclinical stage. Once these problems are resolved, research in the field of MyD88 will advance rapidly. Future research should focus more attention on the cocrystallization of protein and inhibitors as well as the binding sites, so as to develop more effective targeted inhibitors. Meanwhile, the combination of MyD88-targeted therapies and immunotherapy may bring more hope for cancer cure in the near future.

## Abbreviations

CacyBP/SIP	calcycin-binding protein and Siah-1 interaction protein
CI	confidence interval
COX-2	cyclooxygenase 2
CX3CL1	C-X3-C motif chemokine ligand 1
DCs	dendritic cells
DD	death domain
DLBCL	diffuse large B-cell lymphoma
EVs	extracellular vehicles
HGSOC	high-grade serous ovarian cancer
HMGB1	High mobility group box-1 protein
HR	Hazard Ratio
IDO	Indoleamine-2,3-Dioxygenase
IFNs	type I interferons
IFN-γ	interferon-gamma
IKK	IκB kinase
IL-10	interleukin-10
IL-18R	interleukin-18 receptor
IL-1R	interleukin-1 receptor
IL-6	interleukin-6
iNOS	nitric oxide synthase
INT	intermediate domain
IRAK	IL-1R-associated kinase
IRF-7	interferon regulatory factor 7
LGSOC	low-grade serous ovarian cancer
MAPK	mitogen-activated protein kinase
MDSCs	myeloid suppressor cells
MT1-MMP	membrane type 1 matrix metalloproteinases
MyD88	Myeloid differentiation factor 88
NF-κB	nuclear factor-κB
PAUF	pancreatic adenocarcinoma up-regulated factor
PCa	prostate cancer
PD-1	programmed death 1
PD-L1	programmed death ligand 1
Ras/ERK	rat sarcoma virus/extracellular signal-regulated kinase
TAMs	tumor-associated macrophages
TIR	toll interleukin-1 receptor
TLRs	toll-like receptors
TME	tumor microenvironment
TNF-α	tumor necrosis factor α
WM	Waldenstrom macroglobulinemia
WT	wild-type
